# On language acquisition in speech and sign: development of combinatorial structure in both modalities

**DOI:** 10.3389/fpsyg.2014.01217

**Published:** 2014-11-11

**Authors:** Gary Morgan

**Affiliations:** Language and Communication Science, City University London, LondonUK

**Keywords:** sign, acquisition, phonology, classifiers, componential structure

## Abstract

Languages are composed of a conventionalized system of parts which allow speakers and signers to generate an infinite number of form-meaning mappings through phonological and morphological combinations. This level of linguistic organization distinguishes language from other communicative acts such as gestures. In contrast to signs, gestures are made up of meaning units that are mostly holistic. Children exposed to signed and spoken languages from early in life develop grammatical structure following similar rates and patterns. This is interesting, because signed languages are perceived and articulated in very different ways to their spoken counterparts with many signs displaying surface resemblances to gestures. The acquisition of forms and meanings in child signers and talkers might thus have been a different process. Yet in one sense both groups are faced with a similar problem: “how do I make a language with combinatorial structure”? In this paper I argue first language development itself enables this to happen and by broadly similar mechanisms across modalities. Combinatorial structure is the outcome of phonological simplifications and productivity in using verb morphology by children in sign and speech.

## INTRODUCTION

Signed languages have all the levels of complexity and expressive power as spoken languages, they are processed in similar ways by cognitive and related brain networks (Emmorey, 2002) and they can be acquired as native languages by children following the same developmental stages as those identified for spoken language (Petitto, 1997; Chamberlain et al., 2000; Morgan and Woll, 2002; Baker and Woll, 2009; Chen Pichler, 2012). Native signers are a rare group, as only 5–10% of deaf children have deaf parents (Mitchell and Karchmer, 2004). In this paper I focus on two main issues in native sign language acquisition: (1) the relationship between gestures and signs and (2) the emergence of combinatorial structure during language development. To illustrate both issues I use case studies of native signers acquiring BSL. I ague that combinatorial structure distinguishes signs and gestures, and that this difference comes about because of language acquisition mechanisms.

The paper is organized as follows: the first section focuses on signs and gestures and explores how these two forms of semiotic communication are different, based on the presence or absence of conventionalized linguistic representations. In section 2, I describe how children’s development of language leads to combinatorial structure. In section 3, I illustrate the points made in the first two sections by reviewing firstly, the linguistic organization and acquisition of phonology in native signers. The intention is to bring out the broad similarities that exist for phonological development across modalities. Then secondly, in section 4, I describe how spatial utterances in signed and spoken language are organized linguistically and develop in native signers of BSL. This development illustrates the difference between holistic gestures and conventionalized signing with respect to combinatorial structure and also why productivity is important. The paper concludes with some discussion of how research on child sign learners can contribute to a greater understanding of language acquisition in general.

## THE DIFFERENCE BETWEEN SIGNS AND GESTURES IS BASED ON LINGUISTIC ORGANIZATION

The inclusion of the languages of deaf communities into linguistic, psychological, and neurological research has enriched these disciplines and revealed which cognitive processes deal primarily with speech versus those devoted to cross-modality instances of language (Klima and Bellugi, 1979; Pfau et al., 2012). We see that sign languages have a linguistic organization of form and meaning components following traditional ideas of recursion and hierarchical patterning present in all human languages (Chomsky, 1965). These qualities do not appear in gestures which when produced with speech are mostly dependent on the spoken language system (e.g., de Ruiter, 2000). Although quite rare in everyday communication, when gestures are articulated in the absence of speech they take on more language-like properties (Goldin-Meadow et al., 1996). Kendon’s (2004) continuum positions gesture and sign language a distance from each other (McNeill, 1992) but a continuum indicates quantitative rather than qualitative differences (i.e., gesture and sign have a similar and contiguous semiotic underpinning) but this is a contentious point (Singleton et al., 1995). The dis/continuity debate appears in several of the following sections.

It is probably the case that the start of how signs began to evolve comes from homesign systems used before deaf people came together in schools (Senghas et al., 2004; Brentari et al., 2012). In this account, the evolutionary beginnings of specific classes of signs (e.g., classifiers) may have developed from gestures of the surrounding spoken language community (Duncan, 2003; Zeshan, 2003; Van Loon et al., in press).

Although gestures share many similarities with sign languages, on linguistic grounds gestures remain holistic, gradient, and not decomposable (McNeill, 1992; McNeill and Duncan, 2000; Kendon, 2004). In terms of linguistic representation gestures lack the combinatorial structure present in sign language phonology and morphology.

These differences between gestures and signs are important for the field of sign language acquisition where the role of gesture in sign development is still not clearly understood (Volterra and Erting, 1994; Schick, 2004). While the transition between gestures and words in spoken language development has been well documented (Goldin-Meadow, 2003), it has been more difficult to track in signing children because they happen in the same modality. When young hearing children use gestures as they acquire their native spoken language it is transparent to see how the two systems are being used separately or together (Volterra and Erting, 1994). It is less clear how this happens during signed language acquisition or even in the adult system itself (Liddell, 2003). For language development research the debate is about the dis/continuity between gestures and signs. This can be subsumed into a larger question about the modularity or cognitive generality of language (e.g., Petitto, 1987). Returning to the continuum between gestures and sign languages, our question is do children adapt their gestures into sign languages as a gradual and continual process or does sign language acquisition lead to a qualitative reorganization of gesture? The latter implies a profound impact of child language acquisition mechanisms on the structure of language rather than language emerging from gesture as a diachronic process over time. For a similar debate see the role of children in the field of creole genesis (de Graff, 1999). In the following sections I argue that during language development native signers turn communicative gestures into combinatorial grammar.

## CHILDREN’S ACQUISITION OF LANGUAGE LEADS TO COMBINATORIAL STRUCTURE

It has long been suggested that *development* itself drives the change between holistic gestures and combinatorial signs (Newport, 1990). This can be observed in different scenarios. For example by the individual homesigner who creates a conventional system over a life-time (i.e., morphology in Goldin-Meadow et al., 1995), in studies of signed language evolution, where each successive cohort shapes the language from isolated homesigns as a substrate onto a conventional grammar (Senghas et al., 2004; Sandler et al., 2005; Brentari et al., 2012). A still further example is the focus of the current paper which is how the native acquisition of a signed language brings about conventional patterns of phonological and morphological structures. When signing children start to communicate they use communicative gestures, as hearing children do, but at some point in their acquisition of a language they arrive at a system of phonological and morphological conventions. It might be that gestures and sign differ in their linguistic status because native signers are able to create combinatorial language (Singleton and Newport, 2004).

But in one sense conventionalization occurs in every single child who learns a language from their care-givers (e.g., Valian, 2009). Most hearing and deaf native signing children experience optimal input but they still need to arrive at a conventionalized linguistic representation which approximates the adult model. In the bulk of this paper I look at how communication becomes sign language and takes on the linguistic properties underlying a phonological and morphological system. Previous research has documented in detail how gesture and sign differ, however, in this paper I attempt to provide a unifying rationale for how holistic gestures become combinatorial grammar through language development. The argument is that combinatorial structure is an outcome of some well-known mechanisms inherent to first language development: a set of phonological processes in word/sign production (Smith, 1973) and achieving productivity in a morphological system for sign/spoken constructions (Brown, 1973; Tomasello, 1992). I argue that this is why sign languages have the structures they have and come from but are distinguished from gesture.

## THE LINGUISTIC ORGANIZATION OF PHONOLOGY AND SIMPLIFICATION PROCESSES ACROSS SIGN AND SPEECH DEVELOPMENT

The first linguistic descriptions of American Sign Language (ASL) by Stokoe (1960) and Klima and Bellugi (1979) demonstrated a duality of patterning (i.e., control of a phonology and grammar). Individual signs could be broken down into handshape, movement and location parameters, demonstrating systematic phonological structure: a hallmark of all human language (and a contrast to gestures). Later research extended this to other natural signed languages, e.g., British (BSL) or Catalan Sign Language (LSC) and eventually signing was described using mainstream phonological and linguistic theories (e.g., Sandler and Lillo-Martin, 2006).

One part of phonological structure is the existence of minimal pairs, where two lexical items differ by one phoneme only, e.g., [ki] vs. [ti] in “key and tea.” Two signs can also differ in only one parameter, e.g., the BSL signs NAME and AFTERNOON have the same handshape and movement, but the hand moves from the forehead location in NAME and the chin in AFTERNOON. Not all exemplars in each of the sign parameters have the same level of complexity in their internal representations. For example, in the movement parameter a simple exemplar would be in a sign with a straight trajectory of the hand while a sign with a complex movement would be one where the hands move both internally (e.g., by opening and closing) as the hand moves in an arc shape. Brentari (1998) argues these different complexities are related to markedness. This concept has many different interpretations within linguistic theories and is the subject of much debate. While many linguists explicitly define markedness as a grammatical force (i.e., constraints), others have equated it with a notion of sensorimotor complexity (not necessarily specific to language or the grammar). This last interpretation is used in this paper when discussing language acquisition processes.

Brentari (1998) makes clear predictions for which handshapes are marked in ASL, based on the number of features present in the phonological representation, e.g., simultaneous extension and flexion of fingers is one more feature in the representation than one of these movements in isolation. The most complex signs also have the least commonly occurring parameter types in the language. For example, a handshape containing the largest number of selected features in its representation appears in the fewest number of signs. Conversely “unmarked” phonological parameters are those that are phonetically and phonologically simple, as well as frequently occurring in the language. Markedness is important in studies of native signer’s first signs.

### THE EMERGENCE OF PHONOLOGY IS SIMILAR ACROSS MODALITIES

Turning first to the vocal modality, hearing children establish phonological representations for the input extremely early in development (Jusczyk et al., 2002) but in production it is difficult to distinguish actual first words from canonical babbling sounds. First words are often described as unanalyzed or frozen forms (akin to gestures) rather than generated from a system of individual phonemes. Vihman (1995) argued that a phonology for word production emerges once the child has around 50 words in a lexicon and as result of an analysis of contrastive sounds that exist across these words. Further, Vihman (1995) proposed children begin with phonetic templates that they fill in as their phonology develops.

With native signers a similar difficulty in identifying first signs arises, but here we have to deal with the dis/continuity of early hand movements and signs (Petitto, 1987; Volterra and Erting, 1994). Before children first sign they babble with the hands (hand open/closes and palm turns), use ritualized gestures and use pointing. Petitto and Marentette (1991) argued that manual babbling changed over time following a process akin to the transition from variegated to canonical babbling in vocal development. In sign, the movement part of the babbling took on a different cadence as it became part of ASL. Cheek et al. (2001) showed that the earliest parameters appearing in sign babbling were those that would appear first in the child’s initial sign vocabulary. The analog to this in spoken language acquisition would be where children begin with the simplest and most unmarked speech sounds and then gradually extend their repertoires (Vihman, 1995). For example the phoneme /d/ is one of the first speech sounds to be used systematically during the babbling stage and many children acquiring English produce words with these sounds early, i.e., “dada.”

Vihman (1995) proposed that children have an articulatory filter that “screens in” or finds words that are within the child’s phonetic capacity and this means the child may understand/perceive a word but avoid producing it, if it included a speech sound that they cannot yet produce. Ideas of selection and filtering can be traced back to the psycholinguistic models of Smith (1973) where a set of innate and universal processes influenced phonological development. As children are developing their phonological representations the acquisition mechanism implements a set of processes which simplify the sound system and account for the “error patterns” that occur in early language. Smith (1973) described three main types of errors: structural “deletion”; “assimilation” processes and finally systemic “substitution.” Because these processes were labeled universals they can be tested in sign language acquisition (Clibbens and Harris, 1993; Morgan, 2006).

The rest of this section describes how systemic substitution during the acquisition of sign language feeds into the development of the first level of combinatorial structure in signing: phonology. In speech development substitution means the child replaces the adult target sounds not yet mastered, with sounds already part of their productive speech for example producing “tea” instead of “key” and tar instead of “car.” This process is called “fronting” and occurs in typically developing speech until around 4 years. Substitution in children’s first words is linked to the markedness of features (complexity and frequency), as well as the child’s own small rule system at that point in development.

It is also important to note that the earliest phonological forms (handshapes or vocalizations) are the easiest ones for the child to produce motorically. In many sign language acquisition studies with native signers the handshape component is the most difficult element to articulate correctly and substitution is very common (Boyes Braem, 1990; Clibbens and Harris, 1993; Marentette and Mayberry, 2000; Morgan et al., 2007).

Clibbens and Harris (1993) reported that the child in their study who was acquiring BSL, used only the A (fist) and five (spread fingers) handshapes until 1;7, after which she added the G (index point). Clibbens and Harris (1993) proposed that the differences that occur between a child’s production of a sign and the adult target could follow a similar process as those shown for speech and be guided by markedness.

Boyes Braem (1990) proposed a stage model to predict the types of substitutions or simplifications a child might make when acquiring a sign language. As a basic rule if a wrong handshape was used it would come from the same stage or an earlier stage in the model. For example, a child may substitute the five handshape in stage 1 for the B (flat hand fingers closed) handshape in stage 2 of the model. Meier (2005) confirmed this with a larger group of children who followed these same predictable patterns. When they substituted a handshape for an adult target they invariably used one from the first stage of Boyes Braem’s model. These patterns can be related to the idea of systemic processes in Smith’s (1973) model.

As a parallel to Vihman’s (1995) template proposal for spoken language acquisition, Boyes Braem (1990) observed a small set of unmarked handshapes were used by children at the start of their sign acquisition as the initial building blocks of signing phonology. Marentette and Mayberry (2000) and Meier (2005) labeled this first set of handshape phonological “primes.” Primes are invariably unmarked forms, maximally perceptually distinctive (i.e., fully open fingers, fully closed, extended single finger), easiest to produce and occur in high frequency in the adult language. The proposed primes for ASL are the “whole five hand,” the “fist,” and the “index finger” hand and these have been observed in other early child phonologies in other sign languages (e.g., Clibbens and Harris, 1993). Signing children develop a communicative systems based on signs with these prime handshapes. As their vocabulary grows, they attempt to produce more marked handshapes and through well attested phonological processes the output gets re-configured by the child in a systematic way. The claim is that phonological processes feed into the development of componential structure.

Morgan (2006) identified patterns across sign and speech development leading to organization of the phonology. For spoken language development substitutions are typically based on groups of sounds, identified by features. For example, devoicing is a process that may affect all voiced stops. Processes such as devoicing, velar fronting, consonant harmony, or cluster reduction are all different ways to affect groups of sounds (and at the same time, any of these processes may have the result that [t] replaces [d] in a particular instance).

Simplification processes in sign language acquisition are where different primes stand in for visually similar marked handshapes. The idea is that substitutions are through “families” of similar handshapes and far from random. For example the G handshape (index finger) appears as a replacement in all the handshapes that have a “pointing” feature (I, Y, H, F) while not at all as a replacement for the more “fist-like” handshapes (C, W, O, claw 5) where LAX 5 was common (for stills of handsh apes see http://en.wiktionary.org/wiki/Appendix:Sign_language_handshapes).

Thus markedness is dealt with in a similar way by the child at the start of language development across modalities. At the same time there are some modality specific features of sign development. One of these concerns the role of the child’s own visual feedback. Children acquiring spoken languages get full access to auditory feedback of their own voices. However, because many sign locations are not in the signers visual field (e.g., a sign on the signer’s own head) in some cases the child has to produce a sign with less complete feedback. There has not been a lot of research on the role of visual feedback of one’s own signs (Emmorey et al., 2009). However, the developmental data suggests it is useful. Young signers make more self-articulation errors with handshapes that are made at locations in peripheral compared with central vision (Ortega and Morgan, 2010).

A second feature of signing that differs to speech is the size of the major articulators. Young children’s gross movement development influences there articulation of signs. Two characteristics noted in the literature (Meier, 2005) are proximalization (where distal joint articulation in signs changes to joints closer to the body) and sympathy (one handed signs get changed into two handed ones). There do not appear to be comparable phenomena in spoken development.

### INTERIM SUMMARY ON THE ACQUISITION OF PHONOLOGY

Signed and spoken language acquisition is comparable in several ways, with the main overlap for the focus of this review being on how children build componential structure for signs and words. Before children have an established lexicon they use communicative vocal and manual gestures without analyzing sub-lexical contrasts and regularities (Vihman, 1995). Through pressure from a filter/selection model a system of contrasts emerges and in one explanation systemic substitution leads to regularization (Smith, 1973). In signing, the child might look for visual regularities between families of handshapes across the emerging phonology but in both modalities there is an effect of markedness. Children’s sensori-motor limitations lead to strategies for reducing markedness in production and this possibly influences connections between parts of the grammar and the growth of phonological representations (see Newport, 1990 and the “less is more” analogy). Further work is required to test this hypothesis.

In the next section, a mechanism for deriving componential structure is proposed for signing children’s acquisition of spatial classifier constructions following the notion of morphological productivity in linguistic development.

## COMPARISONS OF THE ACQUISITION OF SPATIAL LANGUAGE IN SIGN AND SPEECH

Many psycholinguistic studies make an assumption that there is enough equivalence between the sign and speech modalities to test out theories of language structure and processing. At the same time there do exist aspects that are more divergent. While cross-linguistically there is a very wide range of ways to talk about space and movement, no spoken language articulates words in an actual space like signed languages do with the classifier system. An English sentence such as “the pen is on the table” encodes the semantic components of figure, ground and location in an arbitrary and language specific way (Talmy, 2003). When signers talk about space they use “classifier” constructions whereby the morphological units of the construction can encode entity and spatial semantics simultaneously in real space (see collections in Emmorey, 2003; Morgan and Woll, 2007).

One linguistic description of these constructions proposes a classifier “template” which carries each semantic component attached to each other in a poly-componential verb (Cogill-Koez, 2000). The figure part of the template is the handshape, the path and or motion is shown by the movement of the hand or relative location. Other information can be fitted into the template such as manner, orientation and simultaneity. The convention in BSL and other signed languages is for the ground to be mentioned first, e.g., the sign TABLE is signed in space in front of the signer by moving two flat hands apart at waist height to create a representation of a surface. Then the template gets filled in: figures are encoded using handshapes that represent classes of referents with shared semantic and visual features (e.g., vehicles or long thin objects). The interesting aspect of signed language classifiers is that they use space to talk about space (Emmorey, 2003). This is a device unlike any other in spoken language but does resemble how hearing people use gestures (Schembri et al., 2005; Marshall and Morgan, in press). Originally classifiers were analyzed as poly-morphemic (Klima and Bellugi, 1979; Supalla, 1986) however, recently there is much debate as to how they might incorporate gesture and as such language acquisition data become relevant.

One intriguing comparison concerns signing children’s mastery of the classifier system compared with hearing children. The visual modality might seem much more iconic than words are and would influence the rate and patterning of language development. Here we explore this question using the same two topics described previously: (1) the relationship between gestures and signs and (2) how the child develops combinatorial structure.

In general, spatial language, because of its arbitrariness and cross-linguistic variation is notoriously difficult for children to acquire in spoken languages (Clark, 2004). Although learning to talk about space in spoken languages begins early it continues for several years. Path expressions emerge in the one- and two-word speech of children in different types of languages. Choi and Bowerman (1991) reported that 14–21-month olds who are learning English produce “out,” “up,” and “down” to encode their own paths and “on,” “in,” and “off” for those of objects. By 2 years of age, children use prepositions for encoding topological arrangement of objects, e.g., “on,” “above,” or “below” (Clark, 2004). Projective relations (e.g., behind) are expressed later: in English, Italian, Serbo-Croatian, and Turkish children do not produce “front/back” (e.g., “the ball is in front of the tree”) until about 5 years of age. The use of “left” and “right” to specify the location of one object with respect to another using three-dimensional Euclidean principles appears still later, at about 11 or 12 years (Johnston, 1984). As with other types of morphology the acquisition of spatial language is indexed by productive control over a system (Gentner and Bowerman, 2009). Productivity refers to the acquisition or control of generalizable facts about the system, rather than individualized structures. A child that uses the word “eated” is starting to grasp that the past tense morpheme “ed” can be applied across a class of words in a generalized or productive way (Brown, 1973).

The first studies of the acquisition of classifiers in ASL adopted a poly-morphemic approach and supported this long developmental pattern across modalities (e.g., Schick, 1990). If classifiers are poly-morphemic, children have to grasp the potential to combine semantic contrasts (morphemes encoding an entity “move down,” “turn around,” “be located next to” etc.) across a system of morphological forms (person, vehicle, flat surface etc). It is not a characteristically successful outcome of language acquisition if children remain with only knowledge of isolated constructions. Control of productive knowledge is far more efficient and offers more expressive power (Brown, 1973; Bybee, 2006).

Slobin et al. (2003) reported early use of classifier handshapes and path descriptions in children learning ASL and Sign Language of the Netherlands (SLN). Slobin et al. (2003), describe a deaf child aged 2;8 with non-native SLN input move a fist with thumb and pinkie extended in a downward arc to express the notion “the plane flies down.” Another child exposed to SLN at 2;6 produced two curved spread fingers handshapes and moved them in an upward, slow, zigzag path to show a “balloon drifting away.” An even younger child, at 2;1 exposed to ASL, producing a two handed construction where the less-dominant hand, acts as a ground (representing a chair) with a relaxed spread fingers handshape and the dominant hand with the index and middle finger touching and extended, was placed on top the non-dominant hand to encode the figure-ground meaning “the doll stood on a toy chair.”

Thus the beginning of the grammar might emerge early and even be available to children who are learning a signed language in less than optimal conditions. Slobin et al. (2003) argued these constructions were precocious compared with hearing children acquiring spoken languages and this was because iconicity and gesture gave the child semantic scaffolds which they later develop into a more formal system. An important issue therefore is how early forms linked to iconicity and gesture get put together in a conventionalized and combinatorial way that corresponds to how adult native signers use classifiers in a systematic grammar (de Beuzeville, 2006; Slobin, 2008).

The relevant question is at what point does the child have productive control over combinations of meanings and forms rather than just for individual classifiers (Brown, 1973). In his work on the development of first verb constructions in English Tomasello (1992) proposed children begin to use rules for marking semantic contrast item by item. The verb “island” approach describes children applying rules piece-meal before applying them across constructions (Tomasello, 1992).

Brown (1973) established criterion for attributing productive knowledge in a corpus of utterances where forms are analyzed across different tokens and contexts, e.g., “I walked,” “Teddy talked,” and “Daddy eated,” rather than in isolated examples. By looking for productivity in this way we can more easily start to examine how signed language acquisition becomes a conventionalized system of combinations and how this compares with spoken language development.

Following the verb islands concept a signing child hypothetically might use a classifier handshape for a person in only one context, e.g., FATHER CL-PERSON-GO “daddy goes” and not for any other spatial meanings. Later in development the same handshape CL-PERSON could be combined with a different movement or locative expression to describe a person turning, moving next to, over etc. In this way we could see that the verb islands begin to join up and combinatorial structure is emerging: the handshape begins to be combined with other forms to mark more diverse semantic contrasts and is more productive rather than individuated. Morgan et al. (2008) using Brown’s productivity criterion described classifier forms in the signing of a native signer between 1;6 and 3;0 and how at the start of his language development gestures were used to describe spatial concepts before the classifier system took over.

### COMPONENTIAL STRUCTURE IS DRIVEN BY PRODUCTIVITY

Morgan et al. (2008) described this under-specified use of the classifier system in a case study of native BSL acquisition. They identified gestures, signs and classifiers before looking at how the classifiers developed. Initially they described a usage pattern of sole gestures then gestures combined with parts of the classifier construction template and finally classifiers without gestures. The order of development for spatial language in BSL was:

whole body as the figure > hand as a the figure and real object as ground or *vice versa* > finger tracing the path > conventional classifier construction.

Between 1;10 and 2;6 there were a set of eight meaningful handshapes that the child used in individual utterances or verb islands. They were not being combined with more than one movement/location component and were thus categorized as non-productive forms. During the age 2;6–3;6 the child expanded the number of different handshape and path/location combinations moving from verb islands to a more productive system. Example, at 2;6 the flat hand was used with three different spatial meanings as was the pinkie/thumb handshape. By 3;0 the two finger handshape was used in three different contexts and the same movement/location components of the classifier template were being combined with several different handshapes. Thus different parts of the template were being interchanged which suggests the child has more control over the system (see Morgan et al., 2008 for more details).

It is still a debate as to which mechanisms drive productivity in language acquisition: domain general cognitive mechanisms or language-dedicated rules (Tomasello, 1992; Valian, 2009). For the acquisition of the classier constructions once the child has combinatorial structure they can use the system in a productive way. Structure allows productivity and productivity extends combinatorial structure.

### SUMMARY ON THE ACQUISITION OF SPATIAL LANGUAGE FROM GESTURES TO SIGNS

The development of verb morphemes in spoken languages typically begins on familiar verbs and repetitive contexts before being used with novel items (Tomasello, 1992). The acquisition of classifiers in signing children thus follows a well attested pattern and so productivity is achieved slowly even with available gestures and iconicity. By waiting for productivity we see that the classifier template gets filled in piecemeal. Productivity is signaled when meaning components start to be interchangeable in the template. While spatial language is used in very different ways in signing and speaking children, this developmental path to a combinatorial structure is familiar and predictable.

## LESSONS FROM CHILD SIGN LANGUAGE LEARNERS FOR GENERAL LANGUAGE ACQUISITION THEORY

This paper has presented language acquisition data which documents how mechanisms lead to combinatorial structure in the phonology and morphology of signing. This level of linguistic organization distinguishes signs and gestures. Although they may well be continuous on a spectrum (Kendon, 2004) the acquisition data show that as linguistic systems sign languages are nevertheless subject to typical developmental processes. They are not acquired in a radically different ways to spoken languages but instead conform to how we expect a representational grammar is learned by a child approximating the input and building a conventionalized system.

Findings coming from signing children can inform the general field of language acquisition by firstly emphasizing that linguistic development occurs in universal ways meaning theories that are modality free are preferred. Two more specific observations are also warranted here. We see that native signers use substitution to deal with marked forms by building links between visually related sets of handshapes in their repertoire. These phonological processes can explain why the large group of deaf children who learn sign language late (as they interact with hearing non-signing parents) end up with a different set of phonological abilities when they are adults (Mayberry, 2010). It might be that early reorganization at the sub-lexical level, as a result of maturational limitations, leads children to reap the reward later with more complex phonological representations (Newport, 1990). Secondly, data on spatial language acquisition by signers highlights that children use both holistic gestures and isolated signs initially before arriving at a coherent system with productive linguistic representations. Hearing children acquiring spoken language might also take advantage of the semiotic power of gesture. Early gestures might provide some structure for hearing children to explore meaning and form mappings during language development before their spoken words become part of a productive system. With this in mind continued attention in longitudinal studies of early spoken language development to speech and gesture combinations is worthwhile.

Although native signers are a small number of children their path to the development of componential structure reveals both what is particular about sign language (compared with other visual forms of communication) and what is universal about language acquisition.

## Conflict of Interest Statement

The author declares that the research was conducted in the absence of any commercial or financial relationships that could be construed as a potential conflict of interest.
